# The Order of the British Empire

**Published:** 1918-01-12

**Authors:** 


### THE ORDER OF THE BRITISH EMPIRE.

THE Supplement to the *London Gazette* of January 7 contains the announcement that the King, as Sovereign of the Most Excellent Order of the British Empire, has been graciously pleased to give orders for promotions in and appointments to the Order, dated January 1, for services in connection with the war. The list contains upwards of 2,000 names. This does not include appointments to the Order in respect of services in or for the Dominions and the Colonies, the publication of which is deferred to a later date in order to meet the convenience of the Dominions. The list, dated January 1, includes representatives of the medical profession, science, journalism, finance, National Service, social and welfare work of all descriptions, including many branches affecting women, research, charity, numerous Government departments, Appeal Tribunals, Commandants and donors of hospitals. A large number of awards are made to members of the British Red Cross Society and Order of St. John of Jerusalem, including County and Branch Presidents, Vice-Presidents, Directors, Assistant Directors, Organisers, honorary secretaries and secretaries, quartermasters, and officers in charge of stores, clothing, etc.

The following have received the Order:

MEDICINE, SCIENCE, AND HOSPITALS.

*Knights Commanders* (*K.B.E.*).—Walter Morley Fletcher, Esq., M.D., D.Sc., F.R.S., Secretary of the Medical Research Committee; James Galloway, C.B., Chief Commissioner for Medical Services, Ministry of National Service; Kenneth Weldon Goadby, Esq., Member of the War Office Committee for the Study of Tetanus; Charles Halestaff Kenderdine, Esq., Honorary Secretary and Treasurer, Queen Mary's Convalescent Auxiliary Hospitals, Roehampton; Robert Robertson, Esq., D.Sc., F.R.S., Superintending Chemist, Research Department, Woolwich Arsenal; Lieut.-Col. Alfred Cholmeley Earle Welby, Secretary of the Royal Patriotic Fund Corporation. *Dames Commanders* (*D.B.E.*).—Olive Crofton, Lady Smith-Dorrien, President of the Hospital Bag Fund; Mrs. May Webster, Chairman of the British Women's Hospital Committee. *Commanders* (*C.B.E.*).—Lieut.-Col. J. H. Anderson, Assistant Director of Medical Services, Australian Imperial Force; Benjamin Broadbent, Esq., J.P., of Huddersfield, well known for his work in connection with Child Welfare; Miss Kathleen Burke, the Irish lady who has conducted a successful lecturing campaign in America and elsewhere on behalf of the Scottish Women's Hospitals; Professor Wm. R. Hodgkinson, F.R.S.; Col. Robert D. Rudolf, Consultant in Medicine, Canadian Army Medical Corps. *Officers* (*O.B.E.*).—Major Lionel Oxborrow Betts, Australian Army Medical Corps; Herbert Edmond Cuff, Esq., M.D., F.R.C.S., Principal Medical Officer to the Metropolitan Asylums Board; Miss Margaret Damer Dawson, Commandant of Women's Police Service; the Hon. Essex Eleanora French, Hon. Secretary, Almeric Paget, Military Massage Corps; A. P. Hughes-Gibb, Esq., late Assistant Secretary to the Seamen's Hospital, Greenwich, now Private Secretary to the Food Controller; Andrew Cassels Kay, Esq., Assistant Charity Commissioner; Major Hugh Bennett Lewers, Assistant Director of Medical Services, Australian Imperial Forces; Charles Lupton, Esq., Treasurer and Chairman, Leeds General Infirmary, ex-Lord Mayor of Leeds; Major Thos. McKibbin, Deputy Assistant Director of Medical Services, New Zealand Expeditionary Force; George Reid, Esq., M.D., Medical Officer of Health, Staffordshire County Council; John Robertson, Esq., M.D., Medical Officer of Health, Birmingham; Brevet-Colonel German Sims Woodhead, V.D., Royal Army Medical Corps; Arthur Ferguson Macallan, Esq., F.R.C.S., Director of Ophthalmic Hospitals, Egypt, who has done excellent work in ophthalmic medicine and research; Francis Richard Cassidi, Esq., M.D., Officer in Charge, Transport of First Line Hospitals, Derbyshire.

BRITISH RED CROSS AND ORDER OF ST. JOHN.

*Knights Grand Cross* (*G.B.E.*).—Sir Robert Hudson, Treasurer and Financial Director of the Joint Committee of the British Red Cross Society and the Order of St. John of Jerusalem in England. *Dames Grand Cross* (*G.B.E.*).—Margaret, Baroness Ampthill, C.I., President of the Bedfordshire Branch and Member of Council, British Red Cross Society, head of the Voluntary Aid Detachment Department, Devonshire House; Mrs. Edith Isabel Benyon, President of the Berkshire Branch; Violet Hermione, Duchess of Montrose, President of the Scottish Branch; Mary Elizabeth, Viscountess Northcliffe, Member of the Joint Committee, donor and administrator of Lady Northcliffe's Hospital for Officers. *Knights Commanders* (*K.B.E.*).—Col. Sir Geo. Thos. Beatson, K.C.B., M.D., D.L., V.D., Chairman Scottish Branch; James Cantlie, Esq., M.B., F.R.C.S., Member of Council and of Executive Committee. *Dames Commanders* (*D.B.E.*).—Mrs. Alice May Godman, organiser of Red Cross work; Agnes Lowndes, Lady Jekyll, Head of Stores Department, Order of St. John of Jerusalem; Mrs. Ethel Locke-King, Vice-President North Surrey Division, and Assistant County Director; Mrs. Margaret Ker Pryse-Rice, President Carmarthenshire Branch. A large number of appointments as Commanders (C.B.E.), Officers (O.B.E.), and Members (M.B.E.) have also been made to members of the British Red Cross Society and Order of St. John of Jerusalem.

